# Supplementary material for: MetaRibo-Seq measures translation in microbiomes
Source: Nat Commun. 2020 Jun 29;11:3268. doi: 10.1038/s41467-020-17081-z (PMC7324362; doi:10.1038/s41467-020-17081-z)
Supplement: Supplementary file 10 — Supplementary Data 7 [file 41467_2020_17081_MOESM10_ESM.zip › File2/Confidence_High_Taxonomy/178970_out.krona.html]

Javascript must be enabled to view this page.

members
magnitude
magnitudeUnassigned
count
unassigned
taxon
rank

178970\_out

6

6
superkingdom
2

phylum
6
1239

class
6
186801

186802
6
order

186803
family
6

genus
6
841


SRS012849\_contig\_number\_7771SRS014855\_contig\_number\_contig-100\_711.109499SRS015854\_contig\_number\_contig-100\_997.140186SRS076756\_contig\_number\_22418SRS1041130\_contig\_number\_6016SRS1055022\_contig\_number\_26349
166486
6
species
